# Imaging features of primary type 2 diabetes patients with RR/MDR tuberculosis

**DOI:** 10.3389/fpubh.2026.1834566

**Published:** 2026-05-22

**Authors:** Yuhua Zhao, Qisheng Song, Ying Wang, Kunpeng Wang, Dan Zhang

**Affiliations:** 1 Department of Public Health, Dalian Medical University, Dalian, Liaoning, China; 2 Department of Internal Medicine, Dalian Public Health Clinical Center, Dalian, Liaoning, China; 3 Department of Radiology, Dalian Public Health Clinical Center, Dalian, Liaoning, China

**Keywords:** CT image, multidrug-resistant tuberculosis, multinomial logistic regression analysis, rifampicin-resistant tuberculosis, type 2 diabetes

## Abstract

**Background:**

To the best of our knowledge, imaging features of primary type 2 diabetes mellitus patients with rifampicin-resistant/multidrug-resistant tuberculosis (T2DM RR/MDR-TB) have not been reported in the literature to date. Hence this study investigated the imaging features of primary T2DM RR/MDR-TB patients.

**Methods:**

The clinical data and pulmonary CT findings of 87 primary T2DM RR/MDR-TB patients, 98 primary non-type 2 diabetes mellitus patients with rifampicin-resistant/multidrug-resistant tuberculosis (NT2DM RR/MDR-TB), 86 primary type 2 diabetes patients with drug-susceptible tuberculosis (T2DM DS-TB), and 93 primary pure drug-susceptible TB (DS-TB) patients without T2DM or RR/MDR-TB who were treated at Dalian Public Health Clinical Center from 2018 to 2023 were collected, and the clinical features and imaging differences among the four groups were compared using the chi-square test for categorical variables and the Kruskal-Wallis H test for ordinal or non-normally distributed continuous variables.

**Results:**

According to the results, among the four groups of patients, the NT2DM RR/MDR-TB patients were the youngest (*p* = 6.27e-10). Compared with non-diabetic patients, both T2DM RR/MDR-TB patients and T2DM DS-TB patients had a greater proportion of males (*p* = 1.28e-8) and a greater body mass index (BMI) (*p* = 6.01e-9) but lower albumin levels (*p* = 0.009). T2DM RR/MDR-TB patients presented the highest rates of smoking (*p* = 0.006) and alcohol abuse (*p* = 0.000281). In terms of imaging features, multinomial logistic regression analysis revealed that large nodules (*p* = 0.000239), patchy opacities (*p* = 0.004), non-calcified enlargement of mediastinal lymph nodes (*p* = 0.005), and especially multiple cavities (≥3 cavities) (*p* = 2.21e-8) were high-risk factors for T2DM in RR/MDR-TB patients (*p* < 0.05). As the glycated hemoglobin (HbA1c) levels increased, the incidence of large nodules (*p* = 0.029), multiple cavities (*p* = 0.000030), pleural effusion (*p* = 0.045), and non-calcified enlargement of mediastinal lymph nodes (*p* = 0.001) also increased in the T2DM RR/MDR-TB patients; conversely, the absence of cavities (“0” cavities) (*p* = 0.000009) decreased with increasing HbA1c levels.

**Conclusion:**

Imaging features, such as large solid nodules, patchy opacities, and particularly multiple cavities, are high-risk factors for T2DM in RR/MDR-TB patients. The presence of multiple cavities and extrapulmonary involvement increases with increasing blood glucose levels. Therefore, controlling blood glucose levels may reduce the risk and severity of T2DM RR/MDR-TB.

## 1 Introduction

According to the Global Tuberculosis Report 2025, in 2024, 10.7 million new tuberculosis cases and 1.23 million deaths were reported globally, positioning tuberculosis as the main cause of death due to a single infectious disease worldwide (1).

Given that RR-TB and MDR-TB (TB strains resistant to at least isoniazid and rifampin) patients have similar prognoses, the WHO has included a new drug classification to manage RR and RR/MDR (2, 3). According to the WHO, 400,000 cases (3.7%) of RR/MDR were reported worldwide in 2023 (1). Compared with patients with pure tuberculosis, patients with diabetes and RR/MDR-TB have a greater risk of treatment failure and death (4, 5). Computed Tomography (CT) is one of the diagnostic and therapeutic evaluation methods used for RR/MDR-TB and plays a crucial role in clinical practice. However, while many imaging studies have been conducted on RR/MDR-TB patients (6–12), no research on the imaging features of primary T2DM RR/MDR-TB patients has been reported. Previous studies, including the studies by our research group, have used either a mixture of NT2DM RR/MDR-TB and T2DM RR/MDR-TB patients as subjects, have not clearly specified the subjects, or have been conducted with a mix of primary and re-treated patients without further subgroup analysis, which might have influenced the conclusions of these studies.

This study retrospectively analyzed the clinical and imaging features of 87 primary T2DM RR/MDR-TB patients between 2018 and 2023. The resulting data are expected to serve as an important basis for imaging research on T2DM RR/MDR-TB patients.

## 2 Materials and methods

### 2.1 Ethics statement

This study was reviewed and approved by the ethics committee of Dalian Public Health Clinical Center. All the data, including the records/information of the patients, were de-identified prior to data analysis and analyzed anonymously. Moreover, this study is a retrospective, non-interventional study for which additional informed consent was waived by the ethics committee.

### 2.2 Case definitions

T2DM RR/MDR-TB group: Patients with type 2 diabetes mellitus (T2DM) diagnosed according to the American Diabetes Association criteria reference, i.e., HbA1c ≥ 6.5% (48 mmol/mol), or fasting plasma glucose ≥7.0 mmol/L, or 2-h plasma glucose ≥11.1 mmol/L during an oral glucose tolerance test, or current use of antidiabetic medication. They also had rifampicin-resistant/multidrug-resistant tuberculosis (RR/MDR-TB), defined as *Mycobacterium tuberculosis* resistant to at least rifampicin (and isoniazid for MDR) by phenotypic or molecular drug susceptibility testing (DST).

NT2DM RR/MDR-TB group: Patients without type 2 diabetes mellitus, defined as HbA1c < 5.7% (39 mmol/mol) and fasting plasma glucose <5.6 mmol/L, with no prior diagnosis of diabetes and no antidiabetic treatment. They had RR/MDR-TB using the same DST criteria as group 1.

T2DM DS-TB group: Patients with T2DM (same definition as group 1) and drug-susceptible tuberculosis (DS-TB), i.e., *M. tuberculosis* susceptible to rifampicin and isoniazid (and to all first-line drugs) by DST.

DS-TB without T2DM or RR/MDR-TB group: Patients with DS-TB (same definition as group 3) and without T2DM (same definition as group 2), and without RR/MDR-TB.

### 2.3 Clinical information

The research was conducted at the Dalian Public Health Clinical Center.

The inclusion criteria for patients were as follows: complete clinical data, including demographic information, drug susceptibility testing (DST) results, and CT findings.

Patients with resistance patterns other than RR/MDR-TB and those with comorbid conditions such as chronic obstructive pulmonary disease (COPD), pneumonia, lung cancer, immune system disorders, AIDS, or other underlying systemic diseases were excluded from this study.

During the period between January 2018 and December 2023, a total of 4,229(100%) patients were diagnosed with pulmonary tuberculosis, which was bacteriologically confirmed through smear, culture, or GeneXpert positivity. Of these, 359 (8.49%) had RR/MDR-TB, and after excluding 156(43.45%) re-treatment cases, 203 (56.55%) remained. Another 3,870 (91.51%) were non-RR/MDR-TB; after excluding 909(23.49%) re-treatment cases, 525(13.57%) without DST results, and 303(7.83%) with other resistance, 2,133(55.12%) DS-TB patients remained.

**Figure 1:**
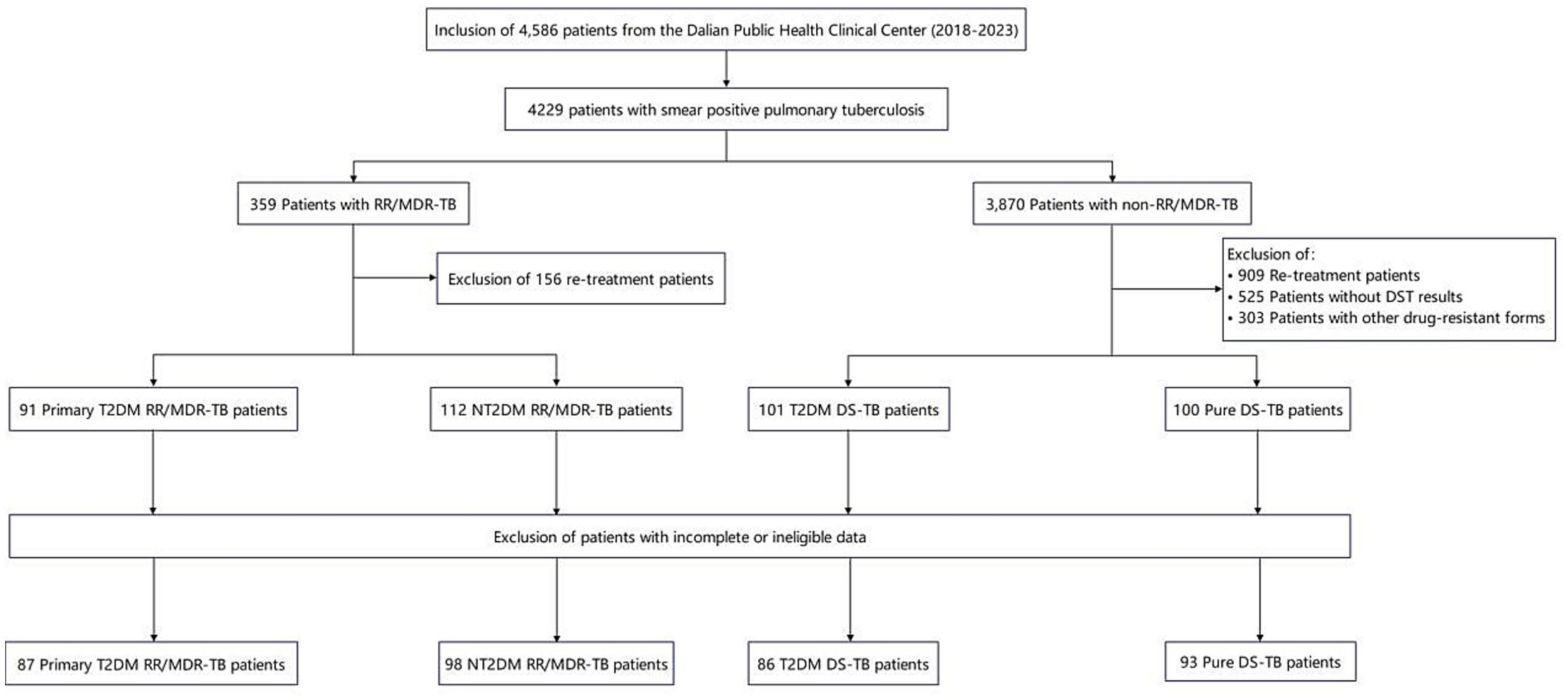
Screening of patients. RR, Rifampicin resistant; MDR-TB, Multidrug resistant tuberculosis; T2DM, Type 2 diabetes; DS, Drug susceptible; DST, Drug susceptibility testing.

Among the remaining 203 patients, 91(44.83%) patients with primary type 2 diabetes RR/MDR-TB and 112 (55.17%) patients were included in the study in chronological order. From the 2,133 DS-TB patients, 101(4.74%) with type 2 diabetes and 100 (4.69%) without were enrolled chronologically. Among these patients, after applying the inclusion and exclusion criteria, the final study cohort comprised 87(95.60%) T2DM RR/MDR-TB, 98(87.5%) NT2DM RR/MDR-TB, 86(85.15%) T2DM DS-TB, and 93(93.0%) pure DS-TB patients. All were HIV-negative. All enrolled patients were HIV-negative (Figure 1).

### 2.4 Research methods

#### 2.4.1 General information

The clinical data of the included patients, including age, gender, smoking history, alcohol abuse history, glycated hemoglobin concentration (HbA1C) at the first diagnosis of TB or RR/MDR-TB, the time intervals between the first diagnoses of DM and TB, body mass index (BMI), and albumin levels, were collected.

#### 2.4.2 CT image acquisition

All the subjects in this study underwent high-resolution computed tomography (HRCT) scans when they were diagnosed. The images were examined and recorded by two radiologists and two clinical experts, and decisions on the CT findings were reached by consensus.

Imaging assessments were performed for the following signs: (1) small nodules < 1 cm in diameter, including trees in buds, branch line nodes, nodules with poorly defined margins, cluster nodules, reticulonodular opacity (combined reticular and nodular opacity), and miliary nodules; (2) large nodules with diameters between 1 and 3 cm; (3) a mass, any circumscribed, mostly solid lesion that measured >30 mm in diameter; (4) patchy opacities, including patchy shadows, irregular flaky infiltration, and ground glass opacities (GGOs); (5) airway damage, including thickening of the airway wall, stenosis or expansion of the lumen, and thickening of the interstitium around the tracheal vascular bundle; (6) the number of cavities, divided into categories of zero cavities, one to two cavities, and three or more cavities; (7) consolidations without cavities, including focal consolidation, segment consolidation, and lobe consolidation; (8) pleural involvement, including pleural thickening or pleural effusion; (9) pericardial involvement, including pericardial thickening or pericardial effusion; (10) enlargement of the mediastinal lymph nodes, including calcified/non-calcified lymph nodes; (11) local calcified lesions; (12) bronchiectasis.

#### 2.4.3 Glycemic control levels

The patients with T2DM RR/MDR-TB were divided into the following three groups based on their HbA1c levels at the initial diagnosis of tuberculosis: HbA1c < 7% (group H1), HbA1c 7–9% (group H2), and HbA1c > 9% (group H3). Pure DS patients were designated the baseline group H0.

### 2.5 Statistical methods

Statistical analyses were performed using SPSS version 26.0. Continuous variables with a normal distribution are expressed as the means ± standard deviations, whereas non-normally distributed continuous variables are presented as medians (P25, P75). Categorical variables are summarized as frequencies and percentages. Intergroup comparisons were conducted using independent *t* tests for normally distributed continuous variables and Mann–Whitney U tests for non-normally distributed variables. Categorical variables were compared via chi-square tests.

Multinomial logistic regression analysis was performed to identify the high-risk factors associated with pulmonary CT manifestations in T2DM RR/MDR-TB patients. Forest plots were generated using GraphPad Prism version 10.1.2 software to visualize the regression results. The correlation between the CT features and glycemic control levels was determined using Spearman’s rank correlation analysis, and correlation heatmaps were constructed using R Studio version 4.3.3.

## 3 Results

### 3.1 Characteristics of the clinical data

Among the four patient groups, those with NT2DM RR/MDR-TB were the youngest, with a median age of 35.50 (26.00, 55.50) years, which was significantly lower than that of the diabetic groups (*p* < 0.05). Compared with non-diabetic patients, diabetic patients were more likely to be male and have a higher BMI but lower albumin levels. Additionally, T2DM RR/MDR-TB patients presented higher rates of smoking and alcohol consumption (Table 1).

**Table 1:** Comparisons of the clinical data of the four groups of patients.

Patient characteristics	T2DM RR/MDR-TB (G1 = 87)	NT2DM RR/MDR-TB (G2 = 98)	T2DM DS-TB (G3 = 86)	DS-TB (G4 = 93)	*p*-value
Age	53.71 ± 11.37	35(26.00, 57.25)	56.98 ± 13.97	49.31 ± 18.31	<0.001^B^
Gender (Male)	77(88.5%)	54(55.1%)	71(82.6%)	52(55.9%)	<0.001^C^
Time interval^A^	5(0.17, 10.00)	–	5(0.58, 10.00)	–	0.537
Smokers vs. nonsmokers	33(37.9%)	18(18.4%)	24(27.9%)	17(18.3%)	0.006^D^
Alcohol abuse	26(29.9%)	10(10.2%)	14(16.3%)	8(8.6%)	<0.001^E^
HbA1C (%)	9.43 ± 1.89	–	9.80 ± 1.99	–	0.210
BMI	22.54 ± 3.66	19.79 ± 2.88	22.55 ± 3.37	20.7(18.59, 23.01)	<0.001^F^
Albumin (g/L)	36.34 ± 6.00	37.32 ± 5.49	36.56 ± 5.75	39.9(34.4, 43.05)	0.009^G^

G1: Group 1, T2DM RR/MDR-TB; G2: Group 2, NT2DM RR/MDR-TB; G3: Group 3, T2DM DS-TB; G4: Group 4, DS-TB. ^A^Time interval between the first diagnoses of DM and TB (years). ^B^G2 < G1, G3, G4, G1 = G3, G1 = G4, G3 > G4. ^C^G1 = G3 > G2 = G4. ^D^G 1 > G2 = G4, G1 = G3, G2 = G3 = G4. ^E^G1 > G2 = G3 = G4. ^F^G1 = G3 > G2 = G4. ^G^G1, G2, G3 < G4, G1 = G2 = G3, G2 = G4.

### 3.2 Distribution of the affected lung lobes

In terms of the distribution of lung lesions, the distributions of lesions in the right middle lobe (*p* = 0.007), left upper lobe (*p* = 0.021), left lingular lobe (*p* = 0.001), and left lower lobe (*p* = 0.014) differed significantly among the four groups of patients. Compared with DS-TB patients, patients with T2DM RR/MDR-TB presented significantly greater involvement of the right middle lobe (*p* = 0.012), left upper lobe (p = 0.012), and left lingular lobe (*p* = 0.001104), along with a greater number of affected lobes, although no significant differences were observed compared with the other patient groups (*p* > 0.05) (Table 2).

**Table 2:** Distribution and comparison of the affected lung lobes among the four patient groups.

Lung lobe	T2DM RR/MDR-TB (G1 = 87)	NT2DM RR/MDR-TB (G2 = 98)	T2DM DS-TB (G3 = 86)	DS-TB (G4 = 93)	*p*-value
RUL	71(81.6%)	77(78.6%)	67(77.9%)	77(82.8%)	0.813
RML	56(64.4%)	42(42.9%)	42(48.8%)	38(40.9%)	0.007^A^
RLL	60(69.0%)	68(69.4%)	55(64.0%)	53(57.0%)	0.253
LUL	70(80.5%)	69(70.4%)	60(69.8%)	55(59.1%)	0.021^B^
Lingula	57(65.5%)	55(56.1%)	50(58.1%)	35(37.6%)	0.001^C^
LLL	56(64.4%)	64(65.3%)	57(66.3%)	43(46.2%)	0.014^D^
Total	370(70.9%)	375(63.8%)	331(64.1%)	301(53.9%)	<0.001^E^

RUL, right upper lobe; RML, right middle lobe; RLL, right lower lobe; LUL, left upper lobe; LLL, left lower lobe. ^A^G1 > G2 = G4, G1 = G3, G2 = G3 = G4. ^B^G1 > G4, G1 = G2 = G 3, G2 = G3 = G4. ^C^G1 = G3 > G4, G1 = G2, G2 = G3, G2 = G4. ^D^G1 = G2 = G3, G1 = G4, G2 = G3 > G4. ^E^G1 = G2 = G3 > G4.

### 3.3 Comparison of the imaging features of the T2DM RR/MDR-TB, NT2DM RR/MDR-TB, T2DM DS-TB, and pure DS-TB cases

Compared with the other three groups, T2DM RR/MDR-TB patients (*p* = 1.17e-10) presented an increased prevalence of cavities, with greater numbers, although no significant difference was observed compared with the T2DM DS-TB patients (*p* = 0.055). The incidences of large nodules (*p* = 0.001038) and patchy opacities (*p* = 0.024) were significantly greater in T2DM RR/MDR-TB patients than in pure DS-TB patients, although the difference was not significant compared with those in the other two groups (*p* > 0.05) (Table 3).

**Table 3:** Comparison of the pulmonary imaging features among the four patient groups.

Pulmonary imaging features	T2DM RR/MDR-TB (G1 = 87)	NT2DM RR/MDR-TB (G2 = 98)	T2DM DS-TB (G3 = 86)	DS –TB (G4 = 93)	*p*-value
Nodules					
Small nodules	87(100%)	98(100%)	83(96.5%)	91(97.8%)	0.084
Large nodules	69(79.3%)	61(62.2%)	60(69.8%)	49(52.7%)	0.002^A^
Mass	7(8.0%)	4(4.1%)	3(3.5%)	8(8.6%)	0.342
Patchy opacities	56(64.4%)	58(59.2%)	50(58.1%)	40(43.0%)	0.024^B^
Airway damage	44(50.6%)	55(56.1%)	36(41.9%)	40(43.0%)	0.168
Cavity					
0 cavities	11(12.6%)	40(40.8%)	16(18.6%)	48(51.6%)	
1–2 cavities	22(25.3%)	25(25.5%)	32(37.2%)	27(29.0%)	
≥3 cavities	54(62.1%)	33(33.7%)	38(44.2%)	18(19.4%)	<0.001^C^
Consolidation					
Focal consolidation	35(40.2%)	46(46.9%)	39(45.3%)	40(43.0%)	0.814
Pulmonary segment consolidation					
0 segments	53(60.9%)	66(67.3%)	53(61.6%)	63(67.7%)	
1 segments	25(28.7%)	23(23.5%)	25(29.1%)	20(21.5%)	
2 segments	4(4.6%)	7(7.1%)	7(8.1%)	8(8.6%)	
≥3 segments	5(5.7%)	2(2.0%)	1(1.2%)	2(2.2%)	0.731
Pulmonary lobe consolidation					
0 lobes	79(90.8%)	90(91.8%)	83(96.5%)	86(92.5%)	
1 lobes	6(6.9%)	6(6.1%)	3(3.5%)	7(7.5%)	
2 lobes	2(2.3%)	1(1.0%)	0(0%)	0(0%)	
≥3 lobes	0(0%)	1(1.0%)	0(0%)	0(0%)	0.470
Pleural involvement					
Pleural effusion	25(28.7%)	16(16.3%)	16(18.6%)	17(18.3%)	0.160
Pleural thickening	24(27.6%)	20(20.4%)	14(16.3%)	14(15.1%)	0.149
Pericardial involvement					
Pericardial effusion	2(2.3%)	1(1.0%)	0(0%)	2(2.2%)	0.632
Pericardial thickening	1(1.1%)	3(3.1%)	1(1.2%)	1(1.1%)	0.733
Enlargement of mediastinal lymph nodes					
Enlargement of mediastinal lymph nodes (non-calcified)	30(34.5%)	15(15.3%)	15(17.4%)	15(16.1%)	0.004^D^
Enlargement of mediastinal lymph nodes (calcified)	7(8.0%)	9(9.2%)	16(18.6%)	21(22.6%)	0.011^E^
Local calcified lesions	6(6.9%)	19(19.4%)	12(14.0%)	30(32.3%)	<0.001^F^
Bronchiectasis	14(16.1%)	32(32.7%)	17(19.8%)	18(19.4%)	0.032

^A^G1 > G4, G1 = G2 = G3, G2 = G3 = G4; ^B^G1 > G4, G1 = G2 = G3, G2 = G3 = G4; ^C^G1 > G2, G1 = G3, G1 > G4, G2 = G3, G2 = G4, G3 > G4; ^D^G1 > G2, G1 > G4, G1 = G3, G2 = G3 = G4; ^E^G 1 < G4, G1 = G2 = G3, G2 = G3 = G4; ^F^G1 = G3 < G4, G1 = G2, G2 = G3, G2 = G4.

With respect to calcified lesions, both calcified mediastinal lymph nodes (*p* = 0.042) and pulmonary calcifications (*p* = 0.000126) were less common in the T2DM RR/MDR-TB patients than in the pure DS-TB patients, although no significant differences were found compared with the other groups (*p* > 0.05). In contrast, non-calcified mediastinal lymphadenopathy was more common in T2DM RR/MDR-TB patients than in non-diabetic TB patients (*p* = 0.004) (Table 3).

Additionally, the statistical analysis conducted using the chi-square test revealed a significant difference in the distribution of bronchiectasis signs among the four groups of patients (*p* = 0.032). However, in the *post hoc* pairwise comparisons with Bonferroni correction, none of the comparisons reached the conventional level of statistical significance, which was attributed to the conservative nature of multiple comparison adjustment. No significant differences among the four groups were noted in the remaining imaging features (*p* > 0.05) (Table 3).

### 3.4 Multinomial logistic regression analysis of the imaging features in T2DM RR/MDR-TB patients

**Figure 2:**
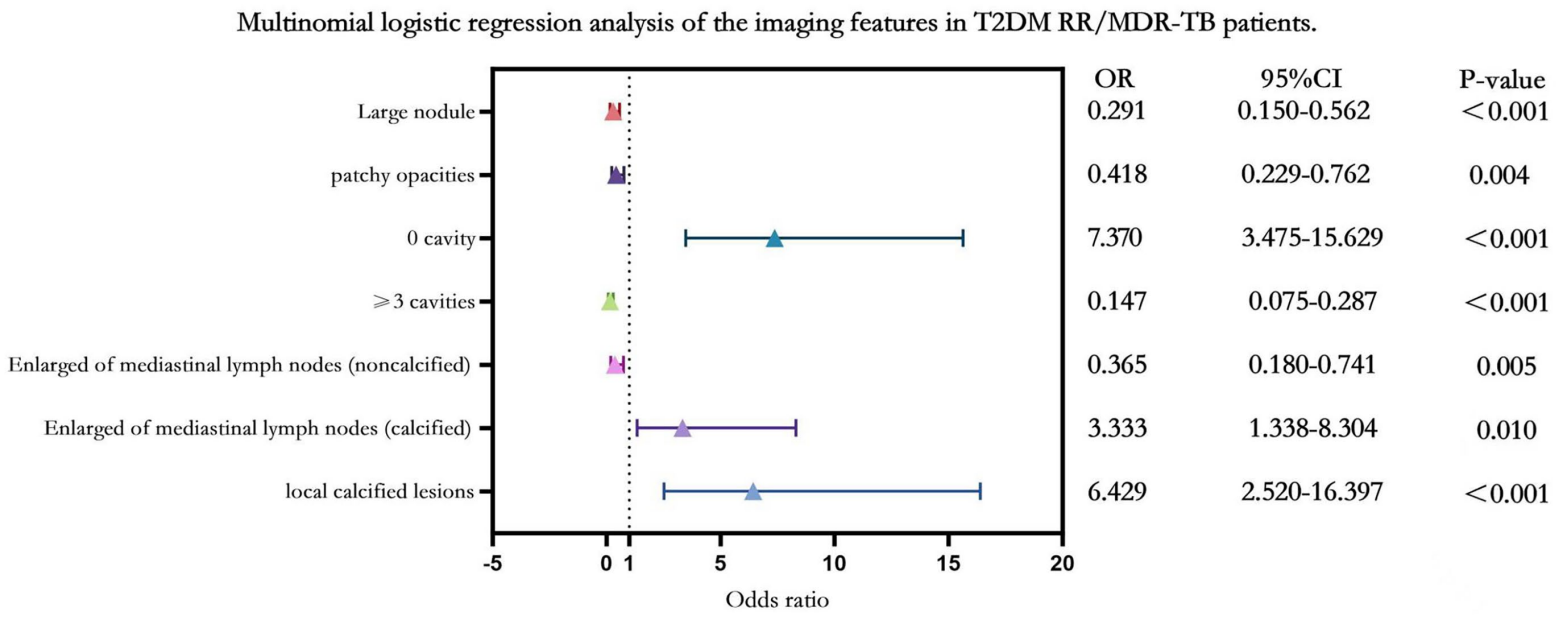
Multinomial logistic regression analysis of the imaging features in T2DM RR/MDR-TB patients.

Before performing multinomial logistic regression analysis, a collinearity diagnosis was conducted on 25 factors: small nodules, large nodules, mass, patchy opacities, airway damage, 0 cavities, 1–2 cavities, ≥3 cavities, focal consolidation, 0 segmental consolidation, 1 segmental consolidation, 2 segmental consolidation, ≥3 segmental consolidation, 0 lobar consolidation, 1 lobar consolidation, 2 lobar consolidation, ≥3 lobar consolidation, pleural effusion, pleural thickening, pericardial effusion, pericardial thickening, Enlarged mediastinal lymph nodes (noncalcified), Enlarged of mediastinal lymph nodes (calcified), local calcified lesions, and bronchiectasis. Tolerance values ranged from 0.629 to 0.969, and all variances inflation factors were below 10, indicating the absence of multicollinearity among the variables. Compared with pure DS-TB patients, T2DM RR/MDR-TB patients presented significantly greater frequencies of large nodules, patchy opacities, non-calcified mediastinal lymphadenopathy, and especially multiple cavities (≥3 cavities) [*p* < 0.05; OR = 6.8 (1/0.147)]. In contrast, the absence of cavities (“0 cavities”) (*p* = 1.92e-7), calcified mediastinal lymphadenopathy (*p* = 0.010), and pulmonary calcifications (*p* = 0.000098) were less common in the T2DM RR/MDR-TB patients than in the pure DS-TB controls (Figure 2 and Table 4).

**Table 4:** Multivariate unordered logistic regression analysis.

Variable	Comparison group	β(*SE*)	OR(95% CI)	Wald X^2^	*p*-value
Large nodules	The model is significant				
	T2DM RR/MDR-TB	−1.236	0.291(0.150–0.562)	13.500	<0.001
	NT2DM RR/MDR-TB	−0.392	0.675(0.379–1.202)	1.778	0.182
	T2DM DS-TB	−0.729	0.483(0.261–0.892)	5.403	0.020
Patchy opacities	The model is significant				
	T2DM RR/MDR-TB	−0.873	0.418(0.229–0.762)	8.105	0.004
	NT2DM RR/MDR-TB	−0.653	0.520(0.293–0.925)	4.952	0.026
	T2DM DS-TB	−0.610	0.543(0.300–0.984)	4.059	0.044
0 cavities	The model is significant				
	T2DM RR/MDR-TB	1.997	7.370(3.475–15.629)	27.117	<0.001
	NT2DM RR/MDR-TB	0.436	1.547(0.873–2.742)	2.230	0.135
	T2DM DS-TB	1.540	4.667(2.368–9.198)	19.801	<0.001
≥3 cavities	The model is significant				
	T2DM RR/MDR-TB	−1.920	0.147(0.075–0.287)	31.304	<0.001
	NT2DM RR/MDR-TB	−0.749	0.473(0.243–0.918)	4.899	0.027
	T2DM DS-TB	−1.194	0.303(0.155–0.591)	12.276	<0.001
Enlarged of mediastinal lymph nodes (non-calcified)	The model is significant				
	T2DM RR/MDR-TB	−1.007	0.365(0.180–0.741)	7.776	0.005
	NT2DM RR/MDR-TB	0.062	1.064(0.488–2.320)	0.024	0.876
	T2DM DS-TB	−0.094	0.910(0.415–1.995)	0.055	0.814
Enlarged of mediastinal lymph nodes (calcified)	The model is significant				
	T2DM RR/MDR-TB	1.204	3.333(1.338–8.304)	6.684	0.010
	NT2DM RR/MDR-TB	1.059	2.884(1.245–6.684)	6.103	0.013
	T2DM DS-TB	0.244	1.276(0.616–2.645)	0.430	0.512
Local calcified lesions	The model is significant				
	T2DM RR/MDR-TB	1.861	6.429(2.520–16.397)	15.171	<0.001
	NT2DM RR/MDR-TB	0.683	1.980(1.020–3.843)	4.075	0.044
	T2DM DS-TB	1.077	2.937(1.388–6.211)	7.945	0.005
Bronchiectasis	The model is significant				
	T2DM RR/MDR-TB	0.224	1.251(0.580–2.701)	0.327	0.568
	NT2DM RR/MDR-TB	−0.703	0.495(0.254–0.963)	4.289	0.038
	T2DM DS-TB	−0.026	0.974(0.465–2.040)	0.005	0.945

### 3.5 Correlation analysis between different glycemic control levels and imaging features in patients with T2DM RR/MDR-TB

**Figure 3:**
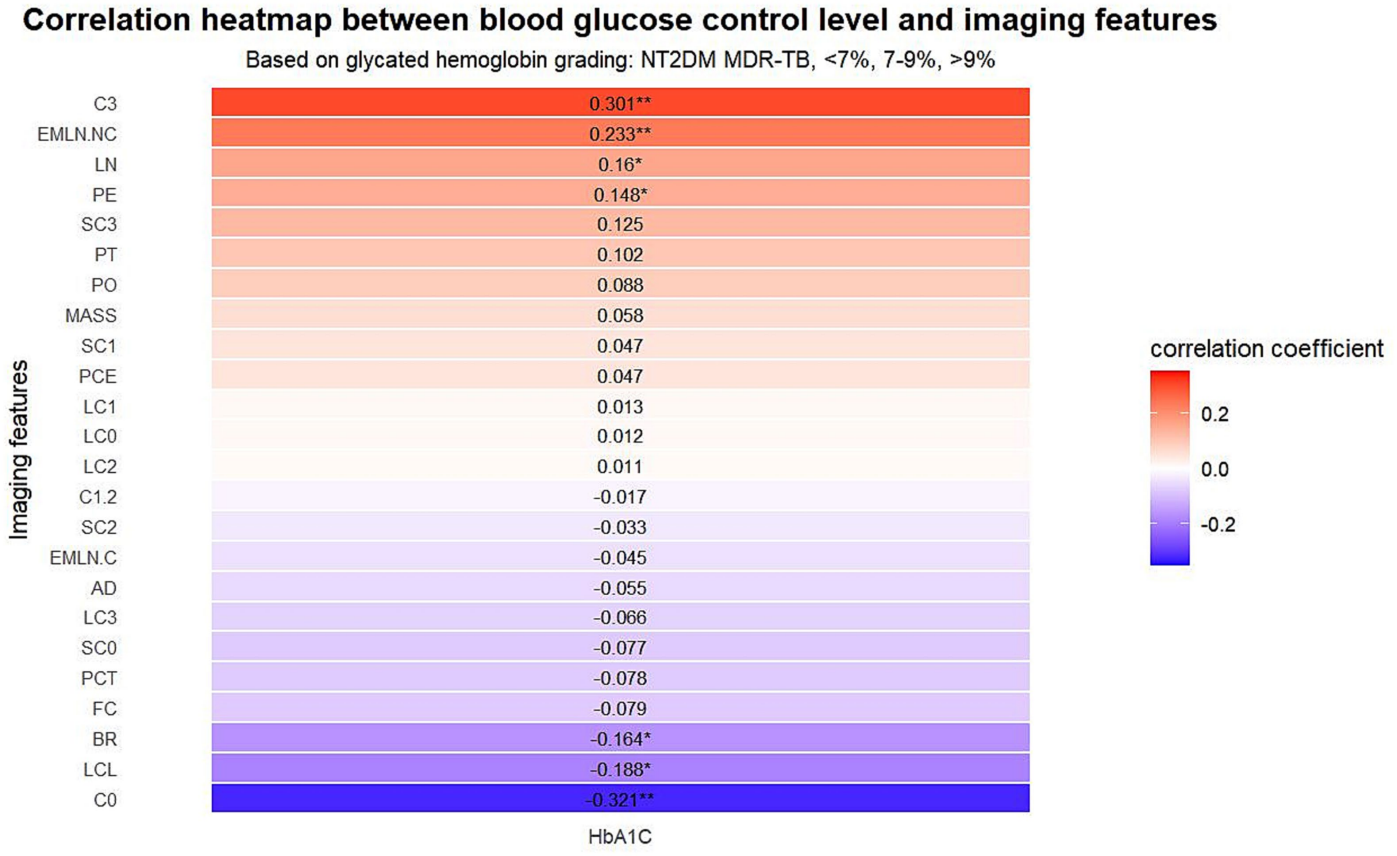
Association of pulmonary CT features with glycemic control in T2DM RR/MDR-TB cases. SN, Small nodule; LN, Large nodule; MAS, Mass; PO, Patchy opacity; AD, Airway damage; C0, 0 cavities; C1.2, 1–2 cavities; C3, ≥3 cavities; FC, Focal consolidation; SC0, 0 lung segment consolidation; SC1, Consolidation of 1 lung segment; SC2, Consolidation of 2 lung segments; SC3, Consolidation of ≥3 lung segments; LC0, 0, lung lobe consolidation; LC1, 1, lung lobe consolidation; LC2, 2, lung lobe consolidation; LC3, ≥3, lung lobe consolidation; PE, Pleural effusion; PT, Pleural thickening; PCE, Pericardial effusion; PCT, Pericardial thickening; EMLN. NC, Enlarged mediastinal lymph nodes (non-calcified); EMLN. C, Enlarged mediastinal lymph nodes (calcified); LCL, Local calcified lesions; BR, Bronchiectasis. * *p* < 0.05, ** *p* < 0.01.

The patients with T2DM RR/MDR-TB were stratified into three groups based on their HbA1c levels: H1 (HbA1c < 7%), H2 (HbA1c 7–9%), and H3 (HbA1c > 9%). A control group of patients with drug-sensitive tuberculosis (H0) was used for baseline measurements. Spearman correlation analysis revealed that with increasing HbA1c levels, the incidence of large nodules (*p* = 0.029), multiple cavities (*p* = 0.000030), pleural effusion (*p* = 0.045), and non-calcified mediastinal lymphadenopathy (*p* = 0.001) also increased, whereas the presence of “no cavity” (*p* = 0.000009) signs decreased (Table 5 and Figure 3).

**Table 5:** Correlation between different levels of blood glucose control and imaging features of T2DM in RR/MDR-TB patients.

Pulmonary imaging features	Hyperglycemia					*p*-value
	T2DM RR/MDR-TB(87)	DS-TB(H0 = 98)	H1(6)	H2(33)	H3(48)	
Small nodules	87	98	6	33	48	––
Large nodules	69	61	4	29	36	0.029
Mass	7	4	1	3	3	0.430
Patchy opacities	56	58	2	20	34	0.236
Airway damage	44	55	3	17	24	0.456
0 cavities	11	40	3	3	5	<0.001
1–2 cavities	22	25	2	9	11	0.822
≥3 cavities	54	33	1	21	32	<0.001
Focal consolidations	35	46	3	14	18	0.288
0 segments	53	66	4	21	28	0.298
1 segment	25	23	2	10	13	0.521
2 segments	4	7	0	1	3	0.652
≥3 segments	5	2	0	1	4	0.089
0 lobes	79	90	6	28	45	0.877
1 lobe	6	6	0	3	3	0.862
2 lobes	2	1	0	2	0	0.882
≥3 lobes	0	1	0	0	0	0.372
Pleural effusion	25	16	1	10	14	0.045
Pleural thickening	24	20	1	8	15	0.168
Pericardial effusion	2	1	0	1	1	0.528
Pericardial thickening	1	3	0	1	0	0.289
Enlargement of mediastinal lymph nodes (non-calcified)	30	15	1	11	18	0.001
Enlargement of mediastinal lymph nodes (calcified)	7	9	2	2	3	0.546
Local calcified lesions	6	19	0	4	2	0.010
Bronchiectasis	14	32	0	5	9	0.026

## 4 Discussion

Despite many imaging-related studies on RR/MDR-TB patients, several issues remain: 1. Several studies have shown that blood glucose levels affect the signs of TB, although the existing studies did not separate diabetic RR/MDR-TB patients from other cases alone during investigation. 2. Previous research, including that by our research group, has not provided a clear indication of whether consolidation with a cavity was classified as pure consolidation or a simple cavity in terms of imaging. 3. Previous studies have not distinguished between primary T2DM RR/MDR TB patients and those with a treatment history. The above problems inevitably affected the study results.

Therefore, for the first time, this study involved an investigation of primary T2DM RR/MDR-TB patients, categorizing all tuberculosis patients into four distinct groups for comparative analysis: T2DM RR/MDR-TB, NT2DM RR/MDR-TB, T2DM DS-TB, and DS-TB.

In this study, based on our previous research, consolidation with a cavity was classified as a cavity, and all the included subjects were primary patients who had not received anti-TB treatment. This eliminated the influence of drugs on imaging signs. Therefore, the research results are expected to be more meaningful for clinical practice.

### 4.1 Analysis of clinical characteristics

In this study, consistent with our previous findings and other studies, non-diabetic patients, particularly those with NT2DM RR/MDR-TB, were relatively younger, which might reflect the fact that type 2 diabetes is predominantly a disease of middle-aged and older individuals. Owing to the higher prevalence of diabetes in older populations, TB patients with diabetes naturally exhibit an older age distribution compared with non-diabetic counterparts. In terms of sex distribution, male predominance, a higher BMI, and lower albumin levels were observed in diabetic tuberculosis patients than in DS TB patients (13). In individuals with diabetes, impaired insulin secretion reduces glucose utilization, leading to increased protein catabolism and subsequent hypoproteinemia (14). This protein deficiency might further compromise cellular immune function, thereby increasing susceptibility to pulmonary tuberculosis (15). Notably, this study revealed that patients with T2DM RR/MDR-TB had significantly higher rates of smoking and alcohol abuse, particularly higher rates of alcohol consumption, than those in the other control groups. Research suggests that smoking suppresses macrophage phagocytic activity against *Mycobacterium tuberculosis* (16), whereas excessive alcohol intake directly damages macrophages and impairs cell-mediated immunity, thereby exacerbating TB progression (17, 18).

### 4.2 Comparison of radiography features

#### 4.2.1 Lung involvement and distribution

The pattern of pulmonary involvement in diabetic patients remains unclear. Some studies have reported an increased frequency of lower-lobe involvement in diabetic patients (19). However, according to the data from the current study (refer to Table 2), no significant differences were observed in lower lobe involvement when T2DM RR/MDR-TB patients and DS-TB patients were compared, and a higher prevalence of left lower lobe involvement was noted in T2DM DS-TB patients than in DS-TB patients. Moreover, no significant difference in right lower lobe involvement was observed across all patient groups.

Notably, compared with DS-TB patients, T2DM RR/MDR-TB patients presented greater involvement in the right middle lobe, left upper lobe, and lingula, along with a greater number of affected lobes. However, no significant differences were observed among the other groups. These radiographic feature disparities in diabetes have been attributed to abnormalities in cell-mediated immunity and polymorphonuclear (PMN) dysfunction (20), including impaired lymphocyte function, reduced monocyte/macrophage counts, and defective chemotaxis and phagocytosis (21). However, no definitive mechanism fully explains the inconsistent findings across studies. Therefore, it was hypothesized that the broader pulmonary dissemination observed in T2DM RR/MDR-TB patients may stem from the higher bacterial load and enhanced invasiveness of drug-resistant strains.

#### 4.2.2 Consolidation and cavitation

Previous research by our group revealed that consolidation, particularly lobar consolidation, was strongly associated with MDR-TB (8). However, the present study, which utilized multinomial logistic regression analysis, revealed that, with the exception of multiple cavities being a high-risk factor for T2DM RR/MDR-TB, consolidation was not included in the regression model. The primary reason for this is, as confirmed in our subsequent research, that consolidation accompanied by cavitation should be radiologically classified as cavitation rather than pure consolidation (22).

#### 4.2.3 Large nodules and patchy opacities

With respect to the imaging features of large solid nodules, consistent with the findings of another study reported by our group (9), patients with T2DM RR/MDR-TB presented a significantly greater prevalence of large solid nodules than those with DS-TB did. Additionally, patchy opacities were more common in the T2DM RR/MDR-TB group than in the DS-TB group. These findings, together with the greater frequency of multiple cavities and greater number of lung fields involved, as mentioned earlier, suggest a more severe and destructive pulmonary pathology in patients with T2DM RR/MDR-TB.

#### 4.2.4 Calcified lesions

This study revealed, consistent with previous findings (8), that patients with T2DM RR/MDR-TB had fewer calcified lesions, including both pulmonary and mediastinal lymph node calcifications, than those with DS-TB. The precise process of pulmonary calcification remains incompletely understood, and the mechanisms underlying calcification due to different etiologies remain unclear (23). One study suggested that osteopontin, which is present in the Golgi apparatus and the secretory granules of macrophages at lesion sites in male Wistar rats, plays a key role in the inflammatory response during tissue wound healing (24). In diabetic patients, the number of macrophages is reduced, as reported previously (21), which may lead to decreased secretion of osteopontin and consequently impaired calcification.

#### 4.2.5 Correlation analysis between blood glucose levels and imaging features

In this study, T2DM RR/MDR-TB patients were categorized into three groups based on their HbA1c levels (<7, 7–9%, and >9%), with DS-TB patients serving as the baseline group. Spearman correlation analysis revealed that as HbA1c levels increased, the incidence of large nodules, multiple cavities, pleural effusion, and non-calcified mediastinal lymphadenopathy also increased. Conversely, the presence of “0 cavities” signs decreased with increasing HbA1c levels, a finding that is consistent with the results reported by Park et al. (25).

The underlying mechanism of these radiographic manifestations likely involves cytokine dysregulation. In 2000, Thomas et al. reported that excessive TNF-*α* and IL-1β are key factors driving tissue necrosis and cavitation in TB patients (26). In 2014, Podell et al. confirmed, using a diabetic guinea pig model, that TNF-α and IL-1β levels were significantly elevated during the chronic phase of infection (90 days post-infection) compared with those in non-diabetic controls (27). In addition, these authors reported upregulated gene expression of interferon-*γ* (IFN-γ), IL-17A, IL-8, IL-10, and monocyte chemoattractant protein-1 (MCP-1) in the lungs and spleens of diabetic TB-infected animals, along with exacerbated neutrophilic inflammation, all of which are closely associated with tuberculous inflammatory exudation and necrosis. Pathological examination in their study further confirmed an increased mycobacterial burden and accelerated disease progression in diabetic guinea pigs. The combined effects of these cytokine alterations and drug-resistant bacterial infection render poorly managed T2DM RR/MDR-TB cases more prone to developing multiple cavitary lesions, with more extensive pulmonary involvement, increased coalescence of nodules into larger nodules, and a greater propensity for extrapulmonary invasion into adjacent structures, such as mediastinal lymph nodes and other structures.

This study has certain limitations that should be acknowledged. First, during the COVID-19 pandemic (2020–2023), some patients might have experienced delayed medical care, potentially leading to more severe and complex radiographic presentations at diagnosis. Second, the pretreatment radiographic findings in primary patients included a mixture of drug-sensitive and drug-resistant bacterial infections, which resulted in overlapping imaging features that may have obscured the typical radiographic patterns of drug-resistant tuberculosis.

In summary, this study demonstrated that in T2DM RR/MDR-TB patients, large nodules, patchy opacities, multiple cavities, and non-calcified mediastinal lymph node enlargement are relatively common and that multiple cavities represent the most frequently observed manifestation. Furthermore, poorer glycemic control is associated with an increased prevalence of multiple cavities, large nodules, and extrapulmonary involvement.

## Data Availability

The raw data supporting the conclusions of this article will be made available by the authors, without undue reservation.

## Glossary

AD: airway damage
BMI: body mass index
BR: bronchiectasis
CT: Computed Tomography
COPD: chronic obstructive pulmonary disease
C0: 0 cavities
C1.2: 1–2 cavities
C3: ≥3 cavities
DS: drug susceptible
DS-TB: primary pure drug-susceptible tuberculosis
DST: drug susceptibility testing
EMLN. NC: Enlarged mediastinal lymph nodes (non-calcified)
EMLN. C: Enlarged mediastinal lymph nodes (calcified)
FC: focal consolidation
GGOs: ground glass opacities
HbA1c: glycated hemoglobin
HRCT: high-resolution computed tomography
LUL: left upper lobe
LLL: left lower lobe
LN: large nodule
LC0: 0, lung lobe consolidation
LC1: 1, lung lobe consolidation
LC2: 2, lung lobe consolidation
LC3: ≥3, lung lobe consolidation
LCL: local calcified lesions
MDR-TB: multidrug resistant tuberculosis
MCP-1: monocyte chemoattractant protein-1
MAS: mass
NT2DM RR/MDR-TB: primary non-type 2 diabetes mellitus patients with rifampicin-resistant/multidrug-resistant tuberculosis
PMN: polymorphonuclear
PO: patchy opacity
PE: pleural effusion
PT: pleural thickening
PCE: pericardial effusion
PCT: pericardial thickening
RR/MDR-TB: rifampicin-resistant/multidrug-resistant tuberculosis
RR: rifampicin resistant
RUL: right upper lobe
RML: right middle lobe
RLL: right lower lobe
SN: small nodule
SC0: 0 lung segment consolidation
SC1: consolidation of 1 lung segment
SC2: consolidation of 2 lung segments
SC3: consolidation of ≥3 lung segments
T2DM: type 2 diabetes
T2DM RR/MDR-TB: primary type 2 diabetes mellitus patients with rifampicin-resistant/multidrug-resistant tuberculosis
T2DM DS-TB: primary type 2 diabetes patients with drug-susceptible tuberculosis
